# What is Lichen planus pemphigoides? A highlight of three cases with discussion of differential diagnosis and suggestion of simple classification guidelines^⋆^

**DOI:** 10.1016/j.abd.2022.08.007

**Published:** 2023-02-06

**Authors:** Reed Maggard, Donna A. Culton, Amy Blake, Paul Googe, Jayson Miedema

**Affiliations:** a University of Washington School of Medicine, Seattle, WA, USA; b Department of Dermatology, University of North Carolina at Chapel Hill, Chapel Hill, NC, USA; c Department of Pathology, University of North Carolina at Chapel Hill, Chapel Hill, NC, USA

Dear Editor,

Cutaneous autoimmune disorders exist on a biological spectrum. A conceptually challenging condition is *Lichen Planus Pemphigoides* (LPP), cases of which appear to share features of bullous pemphigoid and lichen planus. Herein we present three recent cases and emphasize classification as LPP can be made using clinical features in conjunction with histological and immunofluorescence findings. More specifically, classification as LPP can be made in the context of 1) Lichenoid lesions clinically and histologically, 2) Linear staining along the basement membrane zone (BMZ) of IgG and/or C3 on immunofluorescence studies, and 3) Lack of evidence to support another specific diagnosis.

Clinical descriptions of LPP commonly include lichen planus-like lesions with the additional finding of tense blisters and bullae.^1^ The histology is said to be lichen planus-like. Positive immunofluorescence showing deposition along the dermal-epidermal junction is considered a sine qua non-feature. A number of studies have found the autoantigen to be directed against the NC16A subdomain of collagen XVII (BP180).^2^ However, significant heterogeneity in specific target antigen(s) has been documented.^3^, ^4^, ^5^, ^6^

Classification criteria are used to help group conditions for the study.^7^ They are not meant to serve as diagnostic criteria but are often used at a practical level by emphasizing important disease features. Notably, because diagnostic criteria are limited by inherent sensitivity and specificity characteristics, classification criteria are published by the American College of Rheumatology, whereas diagnostic criteria are not. Given the historic controversy associated with LPP, this is a disease for which classification criteria-like guidelines would be clinically useful.

Case 1 was a 55-year-old male with untreated colonic adenocarcinoma who presented with a pruritic rash consisting of violaceous scaly papules and plaques involving the extremities and trunk for several months with more recent blistering (Fig. 1A‒B). Biopsy of a representative lichenoid lesion revealed a brisk lichenoid interface dermatitis histologically consistent with lichen planus (Fig. 1C). Perilesional biopsy for Direct Immunofluorescence (DIF) revealed linear C3 deposition without accompanying IgG (Fig. 1D), cytoid bodies and shaggy, fibrillar fibrinogen deposition at the BMZ. The patient improved on prednisone, without recurrence after the taper.

**Figure 1:**
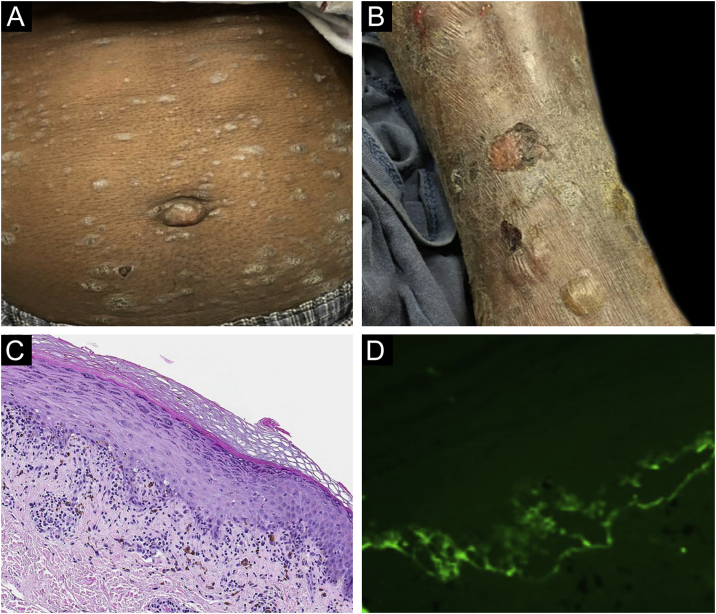
Case 1. (A) Scaly papules and plaques on abdomen. (B) Blistering. (C) Brisk lichenoid interface dermatitis. (D) Linear C3 deposition at the BMZ.

Case 2 was a 29-year-old female who presented with a 6-month history of lichenoid papules and plaques, presumptively diagnosed as lichen planus. At presentation, she was also noted to have scattered vesicles and bullae overlying unaffected and lichenoid skin, distributed on the extremities and trunk (Fig. 2A‒B). The autoantibody workup was negative, and no systemic symptoms were present. A biopsy revealed a robust interface process with features of lichen planus. However, in contrast to typical LP the epidermis was completely detached from the underlying dermis, forming a blistering space histologically (Fig. 2C). DIF showed faint linear IgG, intense linear deposition of C3 (Fig. 2D), and fibrinogen along the BMZ with junctional cytoid bodies. She was started on prednisone and methotrexate with good disease control at follow-up.

**Figure 2:**
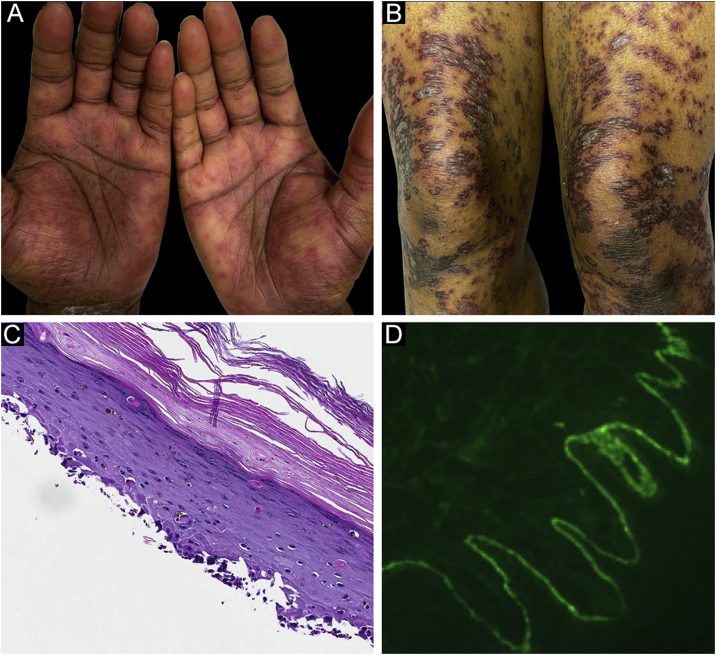
Case 2. (A) Lichenoid lesions with scattered vesicles on hands and (B) thighs. (C) An interface process histologically. (D) Intense linear deposition of C3 along BMZ.

Case 3 was a 68-year-old female who presented with a 2-month history of a scaly pruritic rash. Outside biopsy at the onset of her rash revealed a band-like infiltrate of lymphocytes in the papillary dermis with vacuolar change and necrotic keratinocytes, histologically consistent with lichen planus. One week prior to their presentation in our clinic she additionally developed bullae over the upper extremities and trunk (Fig. 3A‒B). DIF revealed linear C3 deposition at the BMZ without IgG. Indirect immunofluorescence was positive for linear IgG staining localizing to the epidermal side of salt-split skin at a titer of 1:1280. She was treated with triamcinolone wraps, prednisone, and intravenous immunoglobulin with improvement.

**Figure 3:**
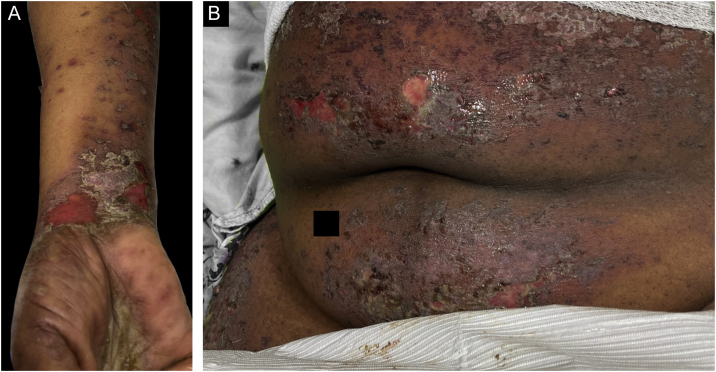
Case 3. (A) Scaly lichenoid and bullous lesions on wrists (B) abdomen.

In all three cases, we were able to exclude traditional lichen planus because of the immunofluorescence findings. Traditional bullous pemphigoid was excluded because of lichenoid histological and clinical findings. None of the cases had other evidence of lupus or bullous lupus erythematosus.

Autoimmune disorders exist on a biological spectrum and often no solitary test has absolute specificity. In the past, authors have noted cases that share features of both lichen planus and bullous pemphigoid and have termed these cases *lichen planus pemphigoid*.

In accordance with our concept of the disease and review of the literature, we emphasize the following classification guidelines: 1) Lichenoid lesions clinically and histologically, precluding classification as routine lichen planus or bullous pemphigoid; 2) Linear staining along the BMZ of IgG and/or C3 on immunofluorescence studies; 3) Lack of diagnostic findings for a separate specific diagnosis, such as lupus erythematosus.

Notably, the above omits the need for more exotic ancillary testing, such as enzyme-linked immunosorbent assay or antigen subtyping.

We choose the term “classification guidelines” in an attempt to parallel other conditions in rheumatology. Challenges in classification are reflected in the American College of Rheumatology’s publication of classification criteria, based on the recognition that rheumatic diseases “tend to be heterogenous in their presentation, course, and outcome and not have a single clinical, laboratory, pathologic, or radiologic feature that could serve as a “gold standard….”^7^

LPP is not a commonly made diagnosis. Zaraa et al. published the largest case review.^8^ They conceptualized the cases as a combination between lichen planus and bullous pemphigoid and emphasize the importance of clinical and histological correlation. Hubner et al. also underscored the importance of careful clinicopathological correlation, especially to exclude other entities.^1^

When presented with similar cases, some clinicians may experience confusion in nosological classification. However, we feel the emphasis of these criteria may help clinicians feel confident in the appropriateness of a diagnosis of lichen planus pemphigoid, when these minimum criteria are satisfied.

## Financial support

None declared.

## Authors’ contributions

Reed Maggard: Approval of the final manuscript version; critical literature review; manuscript critical review; preparation and writing of manuscript.

Donna A. Culton: Approval of the final manuscript version; critical literature review; data collection, analysis and interpretation; intellection participation and/or therapeutic management of studied cases; manuscript critical review; study concept and planning.

Amy Blake: Approval of final version of the manuscript; intellection participation and/or therapeutic management of studied cases; data collection, analysis and interpretation.

Paul Googe: Approval of the final version of the manuscript; data collection analysis and interpretation; intellectual participation and/or therapeutic management of studied cases.

Jayson Miedema: Approval of the final version of the manuscript; critical literature review; intellectual participation and/or therapeutic management of studied cases; preparation and writing of manuscript; manuscript critical review.

## Conflicts of interest

None declared.

## References

[1] Hubner F., Langan E.A., Recke A. (2019). Lichen planus pemphigoides: from lichenoid inflammation to autoantibody-mediated blistering. Front Immunol..

[2] Zillikens D., Caux F., Mascaro J.M., Wesselmann U., Schmidt E., Prost C. (1999). Autoantibodies in lichen planus pemphigoides react with a novel epitope within the C-terminal NC16A domain of BP180. J Invest Dermatol..

[3] Sekiya A., Kodera M., Yamaoka T., Iwata Y., Usuda T., Ohzono A. (2014). A case of lichen planus pemphigoides with autoantibodies to the NC16a and C-terminal domains of BP180 and to desmoglein-1. Br J Dermatol..

[4] Yoon K.H., Kim S.C., Kang D.S., Lee I.J. (2000). Lichen planus pemphigoides with circulating autoantibodies against 200 and 180 kDa epidermal antigens. Eur J Dermatol..

[5] Maoz K.B., Brenner S. (2008). Lichen planus pemphigoides triggered by narrowband UVB, paracetamol, and ibuprofen, with autoantibodies to 130kDa antigen. Skinmed..

[6] Bouloc A., Vignon-Pennamen M.D., Caux F., Teillac D., Wechsler J., Heller M. (1998). Lichen planus pemphigoides is a heterogeneous disease: a report of five cases studied by immunoelectron microscopy. Br J Dermatol..

[7] Aggarwal R., Ringold S., Khanna D., Neogi T., Johnson S.R., Miller A. (2015). Distinctions between diagnostic and classification criteria?. Arthritis Care Res (Hoboken)..

[8] Zaraa I., Mahfoudh A., Sellami M.K., Chelly I., El Euch D., Zitouna M. (2013). Lichen planus pemphigoides: four new cases and a review of the literature. Int J Dermatol..

